# Supplementary figures and images for: Forecasting unprecedented ecological fluctuations
Source: PLoS Comput Biol. 2020 Jun 29;16(6):e1008021. doi: 10.1371/journal.pcbi.1008021 (PMC7375592; doi:10.1371/journal.pcbi.1008021)

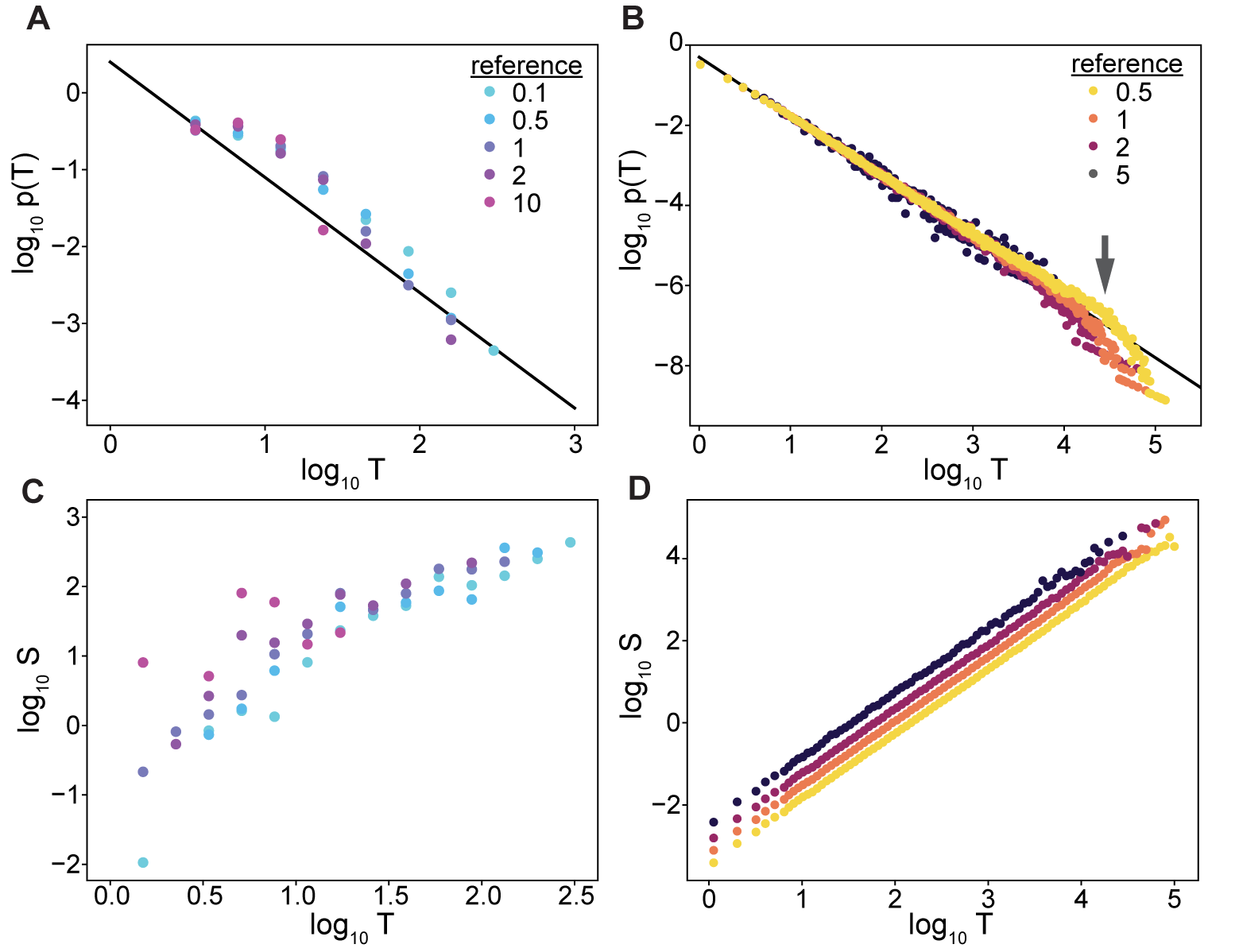

Supplement: S1 Fig — A-B, Probability distribution scaling in herbivorous plankton (A) and linear response model (B) is independent of reference abundance. The references are in the unit of mean abundance. Shifted power-law cutoff (arrow) is due to higher reference that truncates the avalanche duration. C-D, Size-duration scaling exponent independent of threshold abundance. Data in C are noisier at higher references due to the loss of events and limited statistics. Symbol color: same as A and B. (TIF) [file pcbi.1008021.s001.tif]

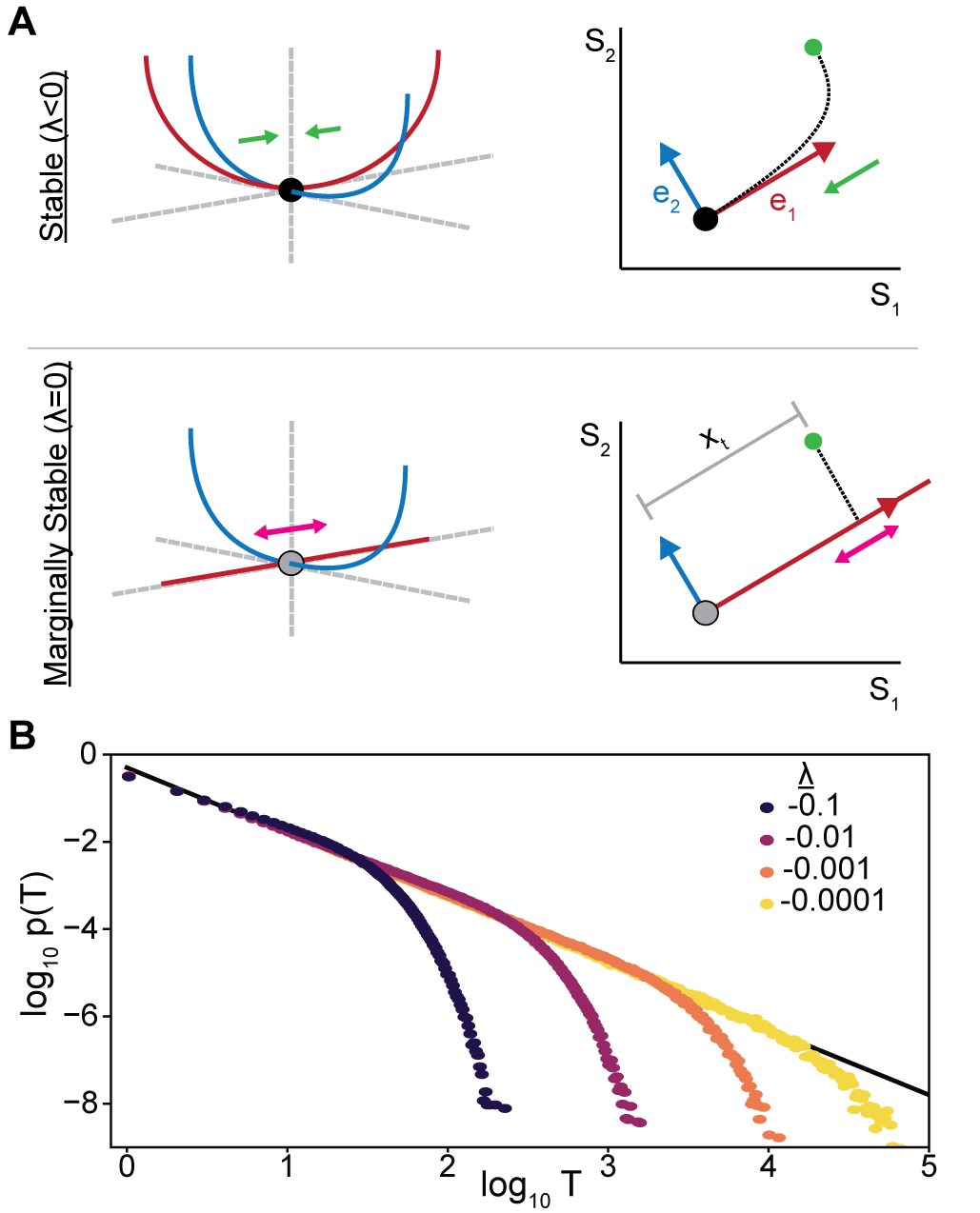

Supplement: S2 Fig — A, Perturbations of ecosystem state (green dot) from a fixed point (black or grey filled symbol) respond along the eigenvectors (red and blue arrows). Top: all eigenvalues are negative. The system is stable and follows a deterministic exponential decay to equilibrium (green arrow). Bottom: one or more eigenvalues approaches zero, creating marginal stability. The deterministic linear response becomes weak relative to stochastic terms (pink arrow), which then drives the system fluctuations along the corresponding eigenvector. B, Probability distribution of avalanche durations in the linear response model (Eq 1) approaches a power law at marginal stability as λ→0. Line: reference slope of 3/2. Simulation run with η~N(0, 0.01). (TIF) [file pcbi.1008021.s002.tif]
