# Supplementary material for: Forecasting unprecedented ecological fluctuations
Source: PLoS Comput Biol. 2020 Jun 29;16(6):e1008021. doi: 10.1371/journal.pcbi.1008021 (PMC7375592; doi:10.1371/journal.pcbi.1008021)
Supplement: S1 Table — Bin average avalanche sizes of individual species from Fig 1D were fit to the equation using linear regression in log-log scale. s.e.: standard error. (PDF) [file pcbi.1008021.s003.pdf]

Group	$\hat{\gamma} \pm s.e.$	$n_{avalanche}$
Harvard Forest Site 1	1.17±0.05	2570
Algae	1.49±0.02	$>10^5$
Mussel	1.52±0.02	$>10^5$
Herbivorous plankton	1.64±0.17	148
Photosynthetic plankton	1.75±0.28	198
Detritivore	1.76±0.17	121
