# Supplementary material for: Forecasting unprecedented ecological fluctuations
Source: PLoS Comput Biol. 2020 Jun 29;16(6):e1008021. doi: 10.1371/journal.pcbi.1008021 (PMC7375592; doi:10.1371/journal.pcbi.1008021)
Supplement: Attachment — Submitted filename: ASE_ReviewerResponse2_final.pdf [file pcbi.1008021.s005.pdf]

Reviewer #1:

We thank again the reviewer for helpful comments. We have addressed all reviewers' comments and the major changes are summarized below and highlighted in red in the manuscript file.

As I understand the linear model, it seems that it is an autoregressive model, that actually seems to produce Brownian motion if lambda goes to zero. I do not understand that such model is so good to predict (for instance lambda 1E-10 in figure 2D should be very close to Brownian motion). Maybe it is because the S-map method on the raw data is performing very badly as it is not suited for this? I would be interested in seeing how another basic prediction method would perform, like tomorrow will be the same as today, or an AR1 model? Is the ASE method also much better than this?

We thank the reviewer for this excellent suggestion. We have implemented an AR1 model with results presented in the new **Fig. 2**. We found that ASE outperforms AR1 almost under every condition. We have modified the text (**lines 170-180 and 465-469**) to include this new result.

Some parts of the methods are really hard for me to follow. What is exactly the role of the standardized avalanche histories in the prediction. How are these included in the S-map method?

S-map requires time series data in order to 'fit' local dynamics. Therefore, to incorporate the information from avalanche scaling into the prediction algorithm, we need to produce time series data based on the scaling laws. For this, we calculated average trajectories of various avalanche sizes and feed them into S-map. This process is described in **Fig. 2A** and discussed in the text (**lines 153-163**).

The division of the data in a training set and a prediction set is not easy to understand. I only found some information in the legend of Figure 3. Why is the training data set length so short (only 10 points)?

We apologize that this point was not clear. We have modified the text to read (**lines 205-210**):

> "To assess forecasting accuracy, we segmented each empirical species dataset from the three ecosystems we study into short time series, each sampling a limited dynamic range. The short training set length allowed us to segment a larger number of independent training sets to evaluate statistical significance when comparing across methods. We used the 50% of segments with smallest magnitudes as training data to predict the 5% largest out-of-sample time points to show that our method is capable of better prediction of fluctuations unprecedented in historical data."

I do not understand the last sentence: "2n statistically independent data sets were segmented .... pvalues are two sided t tests?" What is 2n? 50% are used as training set? (but the length was only 10 points?). What is tested with the t-test?

We have rewritten the figure caption to read (**lines 623-634**):

> "Comparison of S-map forecasts made from raw data (purple) and the hybrid method using both raw and ASE data (orange). To evaluate performance during unprecedented events, we segment empirical data into short segments of lengths specified below. We then use the 50% of segments with lowest average amplitude as training datasets to predict the 5% of time points with largest amplitude. The mean relative error across predictions, $\langle RE \rangle$, is computed for each of these segments and compared between the two methods. Data segment length: 10 time steps for plankton (equivalent of 1 month) and intertidal (equivalent of 10 months) data, 48 steps (equivalent of 1 d) for the forest data. Prediction time: 1 time step for plankton and intertidal data, 4 steps for forest data. Embedding dimension: m = 3 for all species. Bars: median; boxes:

interquartile, whiskers: 5 and 95 percentile; dots: outliers. p-values are computed using a two-sided t-test to compare the mean errors in prediction with raw data only versus the hybrid method. Predictions are made for n training segments in each species, as specified in the figure."
