# Supplementary material for: Forecasting unprecedented ecological fluctuations
Source: PLoS Comput Biol. 2020 Jun 29;16(6):e1008021. doi: 10.1371/journal.pcbi.1008021 (PMC7375592; doi:10.1371/journal.pcbi.1008021)
Supplement: Attachment — Submitted filename: ASE_reviewerResponse_final.docx [file pcbi.1008021.s004.docx]

**Reviewer #1:**

*In this manuscript the authors analyze the distribution of population peaks in some data sets. They call the peaks “avalanches” which is confusing as they suggest that the power law is not due to self-organized criticality (suggesting a build-up of tension), but simply due to random walk. Similar systems are earthquakes and forest fires. They fit a power law to the population sizes and present a way to predict extreme events based on extrapolation.*

We thank the reviewer for helping us improve the manuscript. We agree with the reviewer broadly and the revised manuscript directly addresses all issues raised by the reviewer. All major changes are highlighted in red.

We use the term avalanche to follow the literature in describing a specific type of fluctuations, which are characterized by a set of scaling behaviors as discussed (**line 57-59**). It has been shown that random walks can be one of the *microscopic generative* processes that give rise to avalanche behaviors with power-law exponents consistent with our observations (**ref. 19, 20**). Since the random walk model is irrelevant to our study, we have fully removed the discussion related to random walks. We thank the reviewer for pointing out this potential point of confusion.

*Although the fitted power laws are strikingly similar among different data sets, I am still not convinced of the suggestion that we can use extrapolated power laws to predict extreme events. That is because of several reasons.

1. The theory that all ecosystems are neutrally stable and thus exhibiting random walk is not sell-established in ecology as far as I know. I don’t see how this can explain the tree productivity data.*

We apologize that the original text appeared to claim that all ecosystems are neutrally stable. We have now clarified in the text (**line 122-143**) that the observed avalanche scaling laws in the three empirical systems are consistent with neutral ecology, but the idea of using extrapolation to improve the prediction of ecological fluctuations is not limited to the specific examples we study.

To explore the limit of ASE, we have performed additional analyses. We have added the new **Fig. 2D** which quantifies the performance of predictions using ASE data when the fluctuation dynamics incrementally deviate from marginal stability. In the regime where the system is far from marginal stability, the predictions made with historical data alone outperform the predictions made with ASE data. This finding motivated us to develop a new hybrid version of the forecasting method to integrate both historical and ASE data as the training data set (**Fig. 2A, line 192-199**). We applied this new method to empirical data and found that it improves our prediction accuracy (**Fig. 3**). In addition, we test the limit of this method by making predictions for a chaotic ecosystem (**Fig. 4, line 209-242**). We found that our method is capable of predicting the dynamics effectively. We thank the reviewer for pointing us to this direction, as the new result significantly strengthens the paper.

To answer reviewer’s question on the tree productivity data, we have clarified in the text (**line 394-397**) that marginal stability considers the complete state of the system including species abundances, biomass, and metabolic state. In this case, the carbon flux measures the net metabolic rate within the forest community, which can exhibit fluctuations projected from the dominant eigenvector in the system.

*2. I am not convinced by the tests of the extrapolation tests. If I understand correctly this testing is done in two ways: (1) linear marginally stable model (2) using the low values of the data to predict the peaks. (1) Why do the authors use a LINEAR model that is neutrally stable to simulate species. It doesn’t make sense as the prediction method (S-map) is based on a non-linear model. I could not find the reason why all high dimensional systems should be marginally stable (I could not find that in the cited paper of Stone). That would mean they are (2) It seems obvious that if you predict extreme values from low values that the prediction becomes better if you allow extrapolation. It doesn’t seem a fair test (though I might misunderstood the methods, see next point).*

S-map is a locally weighted linear regression of the time-embedded trajectories. For non-linear dynamics, it assumes that the linear approximation of the embedded dynamics is similar within a local region and makes predictions from nearby historical states. This does not preclude it from making predictions for linear dynamics. We thank the reviewer for helping us to realize that this is indeed confusing for readers who are unfamiliar with the technical details of S-map. We have now clarified the text based on the reviewer’s comments (**line 157-163, 427-433**) and included a new figure panel outlining the method (**Fig. 2A**).

As discussed in the paper by Stone at al. (**ref. 35**), not *all* high dimensional systems are marginally stable, instead, complex systems that can be approximated as a collection of weak random interactions should approach marginally stability, but to various extent. This prediction is based on the statistical distribution of eigenvalues of random interaction matrices. Accordingly, in **Supplementary Fig. 2**, we showed that, within a regime around marginal stability, the avalanche signatures prevail a large dynamic range of fluctuations. This observation motivated us to use the linear response model as a good approximation for near-neutral biology. We thank the reviewer for raising this issue overlooked in the original manuscript, as it is essential to our argument. We have revised the text to make this point clear (**line 122-143**).

With respect to reviewer’s point 2 on extrapolation, S-map makes predictions by identifying nearest neighbors. The linearized dynamics of these neighbors are then extrapolated to the current state. Errors arise when these nearest neighbors are far away and the linear extrapolation becomes inaccurate. In contrast, ASE performs a non-linear extrapolation based on the scaling laws prior to this step. This additional information is what achieves the accuracy improvements. We have made a new figure (**Fig. 2A**) and modified the text (**line 157-163**) to clarify this point.

*3. The figures and method section are very hard to understand. The symbols in the figure axes are not explained (for instance how was Figure 1E constructed and what is on the axes and what does this curve tell me?). Also in the text, not all symbols are explained (T. The structure of the paper is very strange. An important part of the methods are not explained in the method section but in the last part of the result section. It was completely unclear to me what the role is of the linear model when I read the method section. I was searching for details on the ASE method, which was in the result section. I knew the S-map method already, but that would probably also difficult to understand for ecologists that are not familiar with that. Some descriptions in the methods are very condensed and I found it hard to understand what was exactly done. For instance "The normalized avalanche trajectory (′), generated through quadratic interpolation of the converged curve from the linear response model as shown in Fig. 1E, is then scaled according to the size-duration relation Fig. 1D" What makes it extra difficult is that the symbols in the figures are not explained in the legends (later I saw the small figure*

**Fig. 1E** plots the average avalanche shape. We normalized each event by its mean amplitude ($ST^{-1}$) and duration (T) and then calculated the average trajectory of all events. A detailed description for the calculation is now given in both Methods (**line 360-365**) and main text (**line 113-120**). Specifically, the average curve is calculated by averaging the normalized abundance at each normalized time point. Because the data from differently sized events are unevenly sampled after temporal normalization, we use interpolated values of each trajectory to achieve uniform time sampling for averaging. **Fig. 1E** shows that the average path of normalized events converges to a shared shape across communities, which is critical information for ASE as we construct extrapolated events by stretching the average path in both amplitude and duration.

We apologize that the definition of symbols were easy to miss in the original manuscript. *X* denotes the fluctuation amplitude at a given time point; *T* denotes avalanche duration, which is measured as the time between two intersections of the data with the reference level. *S* denotes avalanche size, calculated from the area of the data above the reference. These symbols are defined in the main text at **line 93-96**, and the Methods section at **line 344-347**. We also included an inset in **Fig. 1B**, to aid in defining these terms visually.

We appreciate the suggestion to reorganize the Methods section. We have explained explicitly the role of linear response model (**line 140-143**), added more intuitive descriptions of ASE and S-map in the main text (**line 152-163**), fully rewritten the methods sections on ASE and S-map (**line 410-456**), and added a new figure (**Fig. 2A**) to summarize the method visually. We thank the reviewer for helping us to make this work more accessible.

*4. It was not clear to me how the trajectories were averaged. Why can an average trajectory be used to predict events? The S-map method seems especially suited to predict non-standard trajectories, as it is assumes a response to be state dependent. Moreover I don’t think S-map can predict random walk data sets (as suggested by the marginal stability) as they are fully driven by stochasticity.*

We apologize that this important technical detail of generating average trajectory was not clear in the original text. As mentioned in our reply to reviewer’s last point, this is now clarified in the text, **line 113-115, 360-365**.

ASE extrapolates the average trajectory to arbitrary durations (T) using the empirically confirmed scaling laws. This is useful as it generates an approximate dynamics of large fluctuations that are not sampled in historical data. S-map indeed use non-standard trajectories, but it makes prediction by averaging the historical data within a local region. In other words, using average trajectories is compatible with S-map, and can improve the effectiveness of the averaging step carried out in S-map. Thanks for bringing to our attention this potential point of confusion, which has now been clarified in the text (**line 157-163, 427-433**).

We agree with the reviewer that S-map cannot predict events purely driven by stochasticity. However, it does apply in deterministic systems with stochastic forcing or endogenous random terms (see **ref. 9** and new **ref. 41**). Both near-neutral ecology and the marginal stability model used in this work describe the case where deterministic dynamics still exist, but are relatively weak. We acknowledge that this important distinction was indeed not clear in the original manuscript. Therefore, we have included a new figure, **Fig. 2D**, to systematically explore the regime that ASE can improve forecasting. This new result shows that ASE benefits prediction when stochasticity is significant but the deterministic component is still present, i.e., when $\lambda$ approaches zero but does not equal to zero in the linear response model.

*5. I think there is a physical maximum to the size of the population/productivity peaks. This should be due to resources. The extrapolation method does not seem to account for that, but could predict unrealistically high peaks in biomass/productivity.*

We agree with the reviewer that we do not account for the finite size effect of ecosystem with respect to resources, spatial area, etc., as these parameters cannot always be estimated from historical data. While it remains a theoretical possibility that ASE may make overly large fluctuation predictions, empirically we did not find it to compromise our forecasting accuracy (**Fig. 3**). Unlike the original manuscript where we had taken this point for granted, we now acknowledge this limitation explicitly (**line 282-287**).

*6. Can chaotic dynamics also explain the power law that was found? At least in the used plankton data set, chaotic dynamics were demonstrated. It would be good to check for power law distributions in models that generate such chaotic dynamics (for a model see for instance Dakos et al. 2009 Proc. R. Soc. B (2009) 276, 2871–2880).*

We thank the reviewer for raising this important point. Power law fluctuations are seen in many systems at the transition from linear to chaotic regimes. The ecosystems studied in this paper have been reported to show signs of so-called “edge-of-chaos” state. This includes the plankton data.

To better answer reviewer’s question, we use a model included in the original intertidal work, which is able to drive the dynamics into chaotic regime by tuning a single parameter, $\phi$ controlling the strength of the seasonal forcing function. We chose to analyze this model as an example of chaotic dynamics to avoid confusing readers by introducing another model that generates dynamics of similar nature (e.g., the paper that the reviewer mentioned). We found that chaotic dynamics do not exhibit power law distributions of fluctuation sizes; the dependence of fluctuation size vs. duration also deviates from expectations of avalanche statistics. However, we noticed that even for chaotic dynamics, ASE improves forecasting as it provides a better extrapolation of unobserved fluctuations than the standard S-map method. This new analysis is reported in the new **Fig. 4**.

**Reviewer #2:**

*These authors found that several different ecological datasets showed fluctuations that obeyed scale-free statistics, and that could be modeled using statistical models that were developed for avalanches. Using this realization, one can predict the frequency of extreme events, including those that are outside of any observed events, much more accurately than is possible if one does not account for it.*

*I found the paper to be well-written and interesting, and the research performed well. At least in retrospect, these results don’t seem tremendously profound because it seems reasonable that one could use statistics of past fluctuations to predict future fluctuations, including large ones. I expect that this approach is common in flood forecasting (e.g. the 100-year flood) and in earthquake forecasting. However, I am not a theoretical ecologist, so this may be a new insight to ecology research. In any case, it’s an important one.*

We thank the reviewer for the comments and helping us improve the manuscript. All major changes are highlighted in red.

*As a critique of the work, I’d like to know more about the limits of this approach, showing where it does and doesn’t apply. As part of this, the data sets were chosen carefully to be at the edge-of-chaos and of high quality, with frequent samples taken over a long time period. Would the theory still apply if the ecological system were not at the edge of chaos (e.g. the barnacles in the intertidal data set)? Also, can useful things be said about the data sets that are of lower quality (e.g. the cyclopoids in the plankton data set), or at least predictions made for them?*

We thank the reviewer for raising this important question. We have now explored the applicability of the method in two ways. First, we included a new figure panel (**Fig. 2D**) to test the forecasting accuracy across a range of distance from marginal stability. As the system deviates from marginal stability and deterministic dynamics dominate, predictions made with the historical data alone outperform those made with ASE alone. To address this limitation, we introduce a new version of the forecasting algorithm that incorporates both ASE and the original historical data (**Fig. 2A**, **line 186-200**). We have updated the forecasting performance reported in the new **Fig. 3**. Second, we have examined the applicability of ASE on chaotic dynamics (the new **Fig. 4**). For this, we used the model provided in the original intertidal work, which is able to drive the system dynamics into chaotic regime by tuning a single parameter, $\phi$ controlling the strength of the seasonal forcing function. We found that chaotic dynamics do not exhibit power law distributions of fluctuation sizes; the dependence of fluctuation size vs. duration also deviates from expectations of avalanche statistics. However, we noticed that even for the chaotic dynamics, ASE still improves forecasting as it provides a better extrapolation of unobserved fluctuations than the standard S-map method (**line 209-242**).

We have also now included the prediction results for the ‘low-quality’ data (cyclopoids and barnacles) in **Fig. 3**. Although these datasets do not have sufficient avalanche events for us to extract scaling laws, ASE still significantly (p < 0.05) improves the prediction accuracy. This is now discussed explicitly in the text, **line 204-207**.

*There is no mention as to the cause of the fluctuations here, leading me to initially assume that they arose from intrinsic ecological chaotic dynamics. However, I realized afterward that the primary cause might simply be the weather or other environmental perturbations, especially for the forest and intertidal data sets. Thus, is this fluctuation analysis really an investigation of the statistics of ecological fluctuations, or of environmental fluctuations? If it’s the latter, then it’s still useful for applied work, but it changes the interpretation substantially. Also, it leads to the question of whether weather obeys avalanche statistics, and if that’s the primary driving force for these ecological fluctuations and perhaps others as well***.**

Whether the stochastic driver of dynamics is due to internal fluctuations or random environmental drivers should not change the forecasting algorithm. However, we want to note that the plankton community considered from ref. 28 was grown in a closed laboratory system under largely constant conditions, suggesting that large weather fluctuations are not necessary for the observed power law statistics. Additionally, the stochastic noise in the forcing term (i.e., temperature) in the mussel model is Gaussian distributed, indicating that the power law statistics arise through endogenous feedback through species interactions. We have now discussed this important point explicitly in the text (**line 403-408**).

*In the Linear Response model section, did you use random matrices or non-random matrices? My understanding is that May’s work found that large random matrices had few stable fixed points, and marginal stability for those that were stable, as the text describes here. However, I think more recent work has largely refuted those results because May used random matrices whereas real ecological systems are very non-random, and many of those turn out to be much more stable than originally believed. If this understanding is correct, then this might need to be addressed in your work.*

When modeling the linear response, we assume that the species fluctuations are largely influenced by the response along the dominant eigenvector of a high dimensional system. Therefore, we model the response along a marginally stable axis, as suggested by **ref. 36-37**, so it does not explicitly rely on interaction matrices. This is now clarified in the text (**line 135-136, 394-397**).

*I didn’t really understand the Forecasting method section. Perhaps a figure would help for the explanation, or at least a more detailed explanation of the background material.*

We apologize that the technical specifics of the forecasting method were not clear in the original manuscript. We have now added more intuitive descriptions of ASE and S-map in the main text (**line 148-163**), fully rewritten the Methods section (**line 410-456**), and included a new figure panel (**Fig. 2A**) to explain the method.
